# Crystal Structure of Diedel, a Marker of the Immune Response of *Drosophila melanogaster*

**DOI:** 10.1371/journal.pone.0033416

**Published:** 2012-03-19

**Authors:** Franck Coste, Cordula Kemp, Vanessa Bobezeau, Charles Hetru, Christine Kellenberger, Jean-Luc Imler, Alain Roussel

**Affiliations:** 1 Centre de Biophysique Moléculaire, CNRS UPR4301, Orléans, France; 2 Institut de Biologie Moléculaire et Cellulaire, UPR9022 CNRS, Université de Strasbourg, Strasbourg, France; 3 Architecture et Fonction des Macromolécules Biologiques, UMR6098 CNRS and Aix-Marseille Université, Marseille, France; Natural Resources Canada, Canada

## Abstract

**Background:**

The *Drosophila melanogaster* gene *CG11501* is up regulated after a septic injury and was proposed to act as a negative regulator of the JAK/STAT signaling pathway. Diedel, the *CG11501* gene product, is a small protein of 115 residues with 10 cysteines.

**Methodology/Principal Findings:**

We have produced Diedel in *Drosophila* S2 cells as an extra cellular protein thanks to its own signal peptide and solved its crystal structure at 1.15 Å resolution by SIRAS using an iodo derivative. Diedel is composed of two sub domains SD1 and SD2. SD1 is made of an antiparallel β-sheet covered by an α-helix and displays a ferredoxin-like fold. SD2 reveals a new protein fold made of loops connected by four disulfide bridges. Further structural analysis identified conserved hydrophobic residues on the surface of Diedel that may constitute a potential binding site. The existence of two conformations, *cis* and *trans*, for the proline 52 may be of interest as prolyl peptidyl isomerisation has been shown to play a role in several physiological mechanisms. The genome of *D. melanogaster* contains two other genes coding for proteins homologous to Diedel, namely *CG43228* and *CG34329*. Strikingly, apart from *Drosophila* and the pea aphid *Acyrthosiphon pisum*, Diedel-related sequences were exclusively identified in a few insect DNA viruses of the Baculoviridae and Ascoviridae families.

**Conclusion/Significance:**

Diedel, a marker of the *Drosophila* antimicrobial/antiviral response, is a member of a small family of proteins present in drosophilids, aphids and DNA viruses infecting lepidopterans. Diedel is an extracellular protein composed of two sub-domains. Two special structural features (hydrophobic surface patch and *cis*/*trans* conformation for proline 52) may indicate a putative interaction site, and support an extra cellular signaling function for Diedel, which is in accordance with its proposed role as negative regulator of the JAK/STAT signaling pathway.

## Introduction

The innate immune system is our first line of defense against invading organisms while the adaptive immune system acts as a second line of defense. The innate immune system includes defenses that, for the most part, are constitutively present and ready to be mobilized upon infection. It is not antigen-specific and reacts equally well to a variety of organisms. Historically, the focus of most immunological studies has been on the adaptive response and its hallmarks, namely the generation of a large repertoire of antigen-recognizing receptors and immunological memory. Recently, however, more effort has been expended on understanding the innate immune system, as it became clear that innate immunity is an evolutionarily ancient defense mechanism, which governs the initial detection of pathogens and stimulates the first line of host defense. Invertebrates have proven to be a good model organism to study innate immunity as illustrated by the initial genetic identification of signaling pathways mediating antimicrobial peptide gene expression in Drosophila [1]. The induction of Toll and Imd pathways upon microbial detection leads to the activation of transcription factors of the NF-κb family [2] and then to the expression of hundreds of genes [3], [4]. *Ex vivo* and *in vivo* studies have shown that the JAK/STAT pathway also contributes to inducible gene expression following infection, in particular in the case of viral infections [5]–[8]. The JAK/STAT signal transduction pathway is conserved from insects to mammals and is involved in a wide variety of biological processes such as the cellular proliferation, the stem cell maintenance, the haematopoiesis and the innate immunity responses [9]. This pleiotropic cascade is the principal signaling mechanism for a large array of cytokines and growth factors in mammals.

In Drosophila, this pathway is composed of the JAK kinase Hopscotch and the STAT factor STAT92E and is activated by the receptor Domeless, which is related to the gp130 subunit of the receptor for cytokines of the interleukin-6 family. Domeless is activated by cytokines of the Unpaired family (Upd, Upd2, Upd3). These cytokines are expressed during development but also in response to stress, in particular during infections [9] [10]

In 2002, Perrimon and colleagues have used genome-wide expression profiling to analyze the contribution of different signaling pathways to the innate immune response. They reported that the *Drosophila melanogaster* gene *CG11501* is up-regulated after septic injury and that the JAK/STAT signaling pathway is involved in this induction [5]. This initial study was followed in the Boutros' lab by a genome-wide RNA interference screen in Drosophila cells to identify novel genes involved in the regulation of the JAK/STAT pathway [11]. The *CG11501* gene was identified as a negative regulator of the JAK/STAT signaling pathway in this study, although its precise molecular function is still unknown [11].

The *CG11501* gene encodes a small cysteine-rich protein that we named Diedel. Diedel is 115 amino acids long and contains 10 cysteines. It displays a strong homology with the products of two other *Drosophila melanogaster* genes, *CG34329* and *CG43228*. Diedel does not seem to be conserved in living organisms outside drosophilids apart from the aphid *Acyrtosiphon pisum*. Curiously however, orthologues of this gene are present in the genome of insect DNA viruses of the *Baculoviridae* and *Ascoviridae* families.

In our effort to decipher the molecular mechanisms of the innate immune responses in Drosophila [12]–[15], we determined the crystal structure of the Diedel protein in two crystal forms at 1.15 Å and 1.45 Å resolution, respectively.

## Results

### Structure determination

The Diedel protein crystallized in two crystal forms. Form A crystals belonged to the orthorhombic space group P2_1_2_1_2_1_, with unit-cell parameters *a* = 29.83 Å, *b* = 44.30 Å and *c* = 58.54 Å. Assuming the presence of one molecule in the asymetric unit, the Matthews coefficient (*V*_M_ value) was calculated to be 1.83 Å^3^.Da^−1^ that gave an estimated solvent content of 33% [16]. The structure was solved by the SIRAS method using an iodo derivative and was refined to an R factor of 12.9% and an R-free factor of 15.1% to 1.15 Å resolution. Form B crystals belonged to the orthorhombic space group *P*2_1_2_1_2, with unit-cell parameters *a* = 49.43 Å, *b* = 78.29 Å and *c* = 21.72 Å. Assuming the presence of one Diedel molecule in the asymmetric unit, the *V*_M_ value was calculated to be 1.99 Å^3^.Da^−1^ with an estimated solvent content of 38% [16]. The structure in this crystal form was solved at 1.45 Å resolution by molecular replacement using the form A crystal structure as the starting model and was refined to an R factor of 16.0% and an R-free factor of 19.6%.

### Overall structure of Diedel

The final model for form A includes the entire expressed protein from residue 25 to 115, the 24 first residues being the signal peptide that is cleaved upon exportation, two extra residues (116 and 117) being cloning artifacts, one thiocyanate molecule, one ethylene glycol molecule and 144 water molecules.

**Figure 1:**
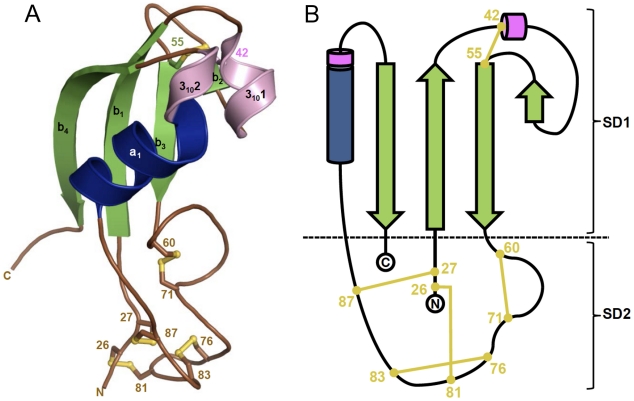
Overall crystal structure of Diedel. (**A**) Overall structure of Diedel. The structure is colored according to its secondary structure elements: α-helices (blue), 3_10_ helices (pink), β-strands (green), and loops (brown). The 10 cysteine residues involved in five disulfide bridges are displayed in yellow and labelled. The N and C terminus residues are mentioned. The figure was generated with PyMOL (http://www.pymol.org). (**B**) Topology diagram of Diedel. The color code for the secondary structure elements is similar to that in figure 1A. Diedel is composed of two sub domains named SD1 and SD2. While SD1 belongs to the ferredoxin-like fold family, SD2 display a quite original fold highly reticulated with four disulfide bridges.

The overall structure shows a relatively elongated shape with approximate dimensions of 60×40×40 Å^3^ (Figure 1A), which can be divided into two sub-domains referred as SD1 and SD2 (Figure 1B). The SD1 sub-domain is made of two segments 29–59 and 90–117, and is composed of a four stranded anti parallel β-sheet (30–35, 48–49, 55–59, 109–113), one α-helix (92–99) and two 3_10_ turns (43–46 and 100–103). The SD2 sub-domain is formed by the first four N-terminal residues (25–28) and by a long loop (residues 60 to 89) connecting the strand β3 and the α-helix of the sub-domain SD1. This domain is highly reticulated with four disulfide bridges; two of them tether the loop 60–89 to the N-terminal residues.

### Difference between Form A and Form B structures

**Figure 2:**
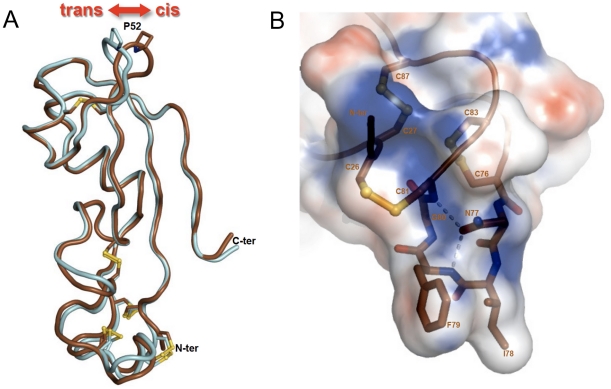
Special features of the Diedel structure. (**A**) Pro52 displays either *trans* or *cis* conformation. The structure obtained with the crystal form A (space group P212121) is colored in brown and that coming for the form B (space group P21212) is colored in cyan. The main structural difference is located in the loop 51–54 and is due to the *cis*-*trans* isomerization of the residue Pro52. (**B**) The loop 76–81 forms a conserved hydrophobic surface patch. The hydrophobic residues Ile78 and Phe79 located on the tip of the loop 76–81 are fully exposed. A network of hydrogen bonds involving the main chain nitrogen of residues 79 and 81 and the OE1 atom of the strictly conserved Asn77 maintains the loop in an extended conformation and contributes in the solvent exposure of the two hydrophobic residues.

The structures solved with the crystal forms A and B have been superimposed with the program Turbo-Frodo. This superimposition indicates that the two structures are nearly identical with 76 Cα among the 92 (82%) of the model displaying equivalent positions in both molecules with distance between the superimposed Cα atoms less than 1 Å. As expected the differences occur in the N- and C-termini and in the tip of the loops. The main structural difference is located in the loop 51–54 with a distance between the Cα atoms of more than 5 Å for the residue 53. The change in the main chain trace is the result of a difference of peptidyl isomerization for the proline 52. Indeed this residue is in *cis* conformation in form A and in *trans* conformation in form B (Figure 2A). The residue P52 is not involved in crystal contact in both crystal forms A and B. Only one distance below 3.5 Å is found between Pro52 and the symmetry related molecules (3.41 Å between Pro52 CB and Gly85 C in form B). Nevertheless positioning the structure found in form A in the crystal packing of form B leads to several clashes (distance below 1.5 Å) between Pro52 and Gly85 of a symmetry related molecule. This is also the case, but in a lesser extend, when putting form B structure in form A crystal packing with few short contacts between Y51 and I48 of a symmetry related molecule. The remaining question is to know whether the different conformations are due to the crystal packing or alternatively if the crystallization leads to the separation of the two coexisting isomers. Unfortunately for the moment we have no data that allow us to answer this question.

### Structural comparisons

A structural similarity search within the Protein Data Bank using the pairwise structural comparison server DALI [17] of the complete Diedel gave no statistically significant global similarities. Searching with the individual subdomains revealed that the overall architecture of SD1 is that of a ferredoxin-like fold (an antiparallel β-sheet covered on one side by two α-helices) with the highest Z-score of 5.4 for a domain of phosphomevalonate (PDB 1K47) with a core RMSD of 2.6 Å. The particularity of Diedel within the ferredoxin-like fold family is the presence of only one α-helix, the first α-helix of the standard fold being replaced by a short 3_10_ helix. Therefore the secondary structure signature of Diedel SD1 domain is β3_10_ββαβ. A Dali search for similar structures to SD2 resulted in no hit.

### Phylogenetic analyses

**Figure 3:**
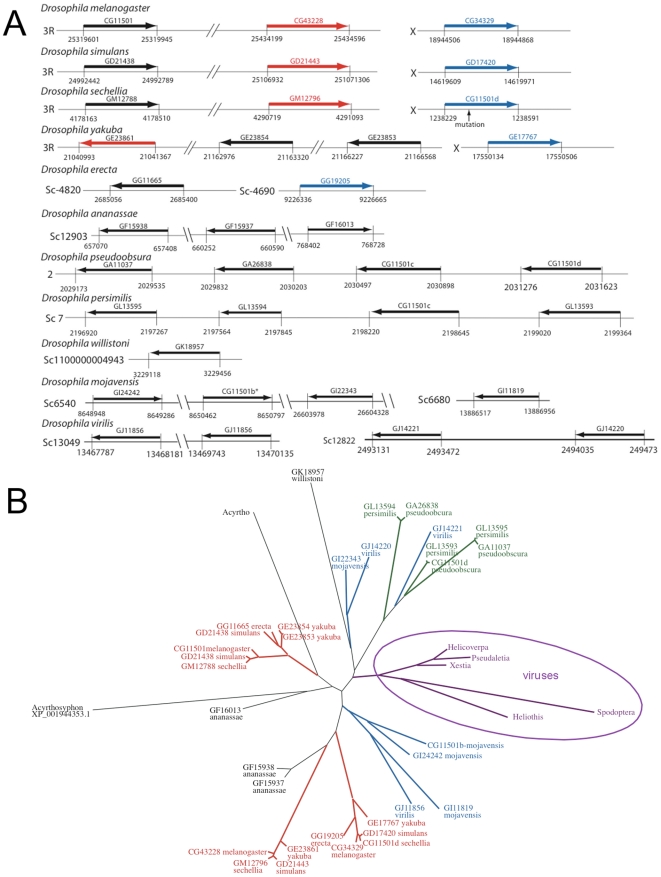
Phylogenetic analysis of *Diedel*. (**A**) Genome localization of *Diedel* and related genes in drosophilids. Genes are named according to Flybase. The asterisk indicates that sequence has been been re-analysed and differs from the annotated one. For each *Drosophila* species are indicated the orientation and the position of the gene or on the chromosome (3R, X, 2) or on a scaffold. The precise position is indicated by the number at the beginning and the end of each base on the sequence given on fly base. When the distances are not too long, the position respect a scale (for pseudoobscura, persimilis and virilis). In the case of virilis, the gene GJ11856 is duplicated. No new ID has been proposed. (**B**) Phylogeny of Diedel-related molecules. The proteins are named according to Flybase for Drosophila species. IDs of the viral molecules can be found in Text S1. In red the melanogaster subgroup, in green the obscura group, in blue the repleta and the virilis groups, in purple the sequences from viruses. The sequences are listed in Text S1.

The genome of *D. melanogaster* contains two other genes coding proteins homologous to Diedel, namely *CG43228* (*Diedel-2*) and *CG34329* (*Diedel-3*). *Diedel* and *CG43228* are located very close to each other on the chromosome 3R whereas *CG34329* is located on the X chromosome. The synteny of these three genes is conserved among the melanogaster group (*D. melanogaster*, *D. similans*, *D. sechellia*, *D. yakuba* and *D. erecta*) with some exceptions: (i) a fourth gene is present in *D. yakuba*; (ii) the gene located on the X chromosome in *D. sechellia* displays a mutation making it inoperative (unless it is a sequencing error); and (iii) only two genes have been retrieved in *D. erecta*, for which the annotation is not fully completed (Figure 3A). Interestingly, phylogenetic analyses indicate that these three genes originated between the speciation events in the melanogaster group (Figure 3B). A similar situation is found for the obscura, the replete and the virilis groups. This suggests that the common ancestor of drosophilids had a single *Diedel* gene, which was subsequently duplicated independently in the different *Drosophila* groups.

Diedel molecules are not found in the sequenced genomes of other insects, with the exception of the pea aphid *Acyrthosiphon pisum*. Surprisingly, the two *A. pisum* genes encoding Diedel-like molecules are more closely related to the *Diedel* genes found in the melanogaster group than those found in the other drosophilids. Apart from *Drosophila* and pea aphid, *Diedel* related sequences were only identified in some insect DNA viruses of the Baculoviridae (*Pseudalatia unipuncta* granulovirus, *Helicoverpa armigera* granulovirus and *Heliothis armigera* granulovirus) and Ascoviridae (*Spodoptera frugiperda* ascovirus) families (Figure 3B). Strikingly all these viruses infect lepidopterans and not dipterans. The independent acquisition of *Diedel* genes in two distinct families of DNA viruses [18] strengthens the connection between Diedel and the field of infectiology.

**Figure 4:**
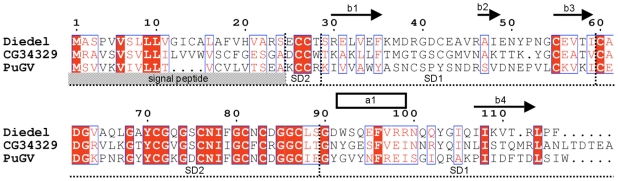
Amino acid sequence alignment of Driedel and other homolous proteins. The Diedel protein of *Drosophila melanogaster* was aligned to homologous gene products from *Drosophila melanogaster* (CG34329) and *Pseudalatia unipuncta* granulovirus (PuGV). The numbering is that of Diedel in this study. Secondary structure elements, *i.e.* strands and helices, are indicated below the sequences as arrows and rectangles, respectively. Conserved residues are boxed, and strictly conserved residues are shown in white with a red background. Note that the level of sequence identity is much more higher in the SD2 sub-domain than in the SD1 sub-domain. The figure was generated with ESPript [36].

Figure 4A shows the sequence alignment of members from three subfamilies of Diedel-related molecules, namely, Diedel, CG34329 and the viral homolog encoded by the genome of the *Pseudalatia unipuncta* granulovirus. By contrast with the SD1 domain that displays sequence divergences, the SD2 domain is highly conserved in the three subfamilies. Indeed 22 out of 29 residues display high level of conservation. Among them 9 are strictly conserved. Interestingly the loop 76–81 that is highly conserved exposes hydrophobic residues (Ile78-Phe79 in Diedel). The conformation of the loop 76–81 seems not to be induced by the crystal packing as the residues of the loop are not involved in crystal contact and the conformation remains similar in the two crystal forms. The peculiar position of these hydrophobic residues at the tip of the loop (figure 2B) may suggest a possible protein-protein interaction site, mediating association to a cellular receptor or a microbial/viral molecule.

## Discussion

We have solved the crystal structure of Diedel in two crystal forms at 1.15 Å and 1.45 Å resolution, respectively. The structure is composed of two subdomains, one of these belonging to the ferredoxin family fold. The other one displays a particular fold highly reticulated by three disulfide bridges and not found in any other structure of the protein data bank. The name Diedel, which is the german translation of Tweedle, comes from the presence of two domains in such a little protein. Indeed, Tweedle-dum and Tweedel-dee are two little twins in Alice's adventures in wonderland by Lewis Carroll. The lack of functional data for Diedel impedes a structure-function relationship analysis. However several molecular and structural features may be pointed out. Diedel is an extracellular protein, which presents a high level of stability certainly due to the presence of five disulfide bridges. Protein disulfide bonds are formed in the endoplasmic reticulum of eukaryotic cells upon exportation. All the sequences of Diedel-like proteins from Drosophila and aphids and most of viral sequences display a signal peptide for exportation. To the best of our knowledge, there is only one exception in the sequence from Xestia nigum granulovirus. In this case, the disulfide bonds may be formed in the intracellular medium upon the control of a sulfhydryl oxidase, an enzyme present in baculoviruses [19]. Diedel overall structure somewhat resembles that of certain cytokines of the CC or CXC chemokine families with a β-sheet covered by an α-helix. The conservation of several hydrophobic residues at the molecular surface may be the indication of a protein-protein interface. The existence of two conformations for the proline 52 may also have some significance. Indeed, an increasing number of reports indicate that peptidyl prolyl *cis*-*trans* isomerisation can play the role of a molecular switch in numerous physiological mechanisms [20]. In some proteins, proline isomerization may confer conformer-specific properties to a native protein fold by modulating the features its molecular surface [21]. All these features suggest an extra cellular signaling function for Diedel, which would be in accordance with its proposed role as negative regulator of the JAK/STAT signaling pathway. The determination of the tridimensional structure of Diedel paves the way for further studies at both the functional and structural levels to assess its role in the immune response of *Drosophila*.

## Materials and Methods

### Cloning, expression, and purification

Diedel overexpression and purification were performed as using protocols previously described [22]. Briefly, the amplified cDNA fragments of the *CG11501* gene were subcloned into the *Spe*I/*Mse*I site of the pMT/V5-His A plasmid (Invitrogen). The recombinant protein with its own signal peptide was overexpresssed by induction of S2 cells with CuSO_4_ at a cell density of 3.10^6^ cells.ml^−1^. After five days, cells were aseptically centrifuged, resuspended in fresh medium and induced again for five days. Up to ten inductions could be done using the same cells. The insoluble material was removed from the harvested medium by centrifugation at 4°C and 4,000 g for 5 min. The supernatant was clarified by filtering through 0.45 mm filter and loaded onto a chelating column (Chelating sepharose FF, GE Healthcare) equilibrated in 20 mM Na-phosphate pH 7.4 and 500 mM NaCl. Diedel was eluted with equilibration buffer containing 200 mM imidazole. Fractions containing Diedel were pooled, concentrated and buffer-exchanged into 20 mM Hepes pH 7.2 and 150 mM NaCl using a Millipore Ultrafree-15 spin concentrator with a 3 kDa molecular-weight cutoff. The V5-His tag used for purification was removed by an overnight trypsinolysis at 4°C using a trypsin∶Diedel (w/w) ratio of 1∶200. The cleavage product was then loaded onto a HiLoad 16/60 Superdex 75 gel-filtration column (GE Healthcare) equilibrated with 20 mM Hepes pH 7.2, 150 mM NaCl and 0.05% NaN_3_. The purified protein showed a single band with a molecular weight around 10,000 Da in SDS-PAGE as expected after cleavage. Maldi-Tof mass spectrometry analysis showed a main peak at 10,565 Da corresponding to a cleavage site between residues L119 and E120 of the V5-His tag.

### Crystallization and data collection

The JCSG+ crystal screen (Molecular Dimensions) was used to search for the initial crystallization conditions using the hanging-drop vapor-diffusion method at 20°C with a protein concentration of 6 mg.ml^−1^ in 20 mM Hepes pH 7.2, 150 mM NaCl, 0.05% NaN_3_. After several days, two crystal forms A and B appeared in conditions 14 and 4, respectively.

The thin plate-like crystals stacked on each other of form A appeared in 200 mM NaSCN, 20%(v/v) PEG 3350. Subsequent optimization of parameters such as precipitant and protein concentrations, pH range and additives led to thicker plate-like polycrystals with a final well solution of 50 mM CH_3_COONa pH 5.5, 200 mM NaSCN, 18–25%(v/v) PEG 3350. Crystals grew within one week to maximum dimensions of 0.5×0.4×0.08 mm. Form B crystals grew in 20 mM CaCl_2_, 100 mM CH_3_COONa pH 4.6, 30% (*v/v*) MPD and subsequent optimisation gave crystals of 0.3×0.3×0.05 mm after one week.

Single crystals of form A were separated from each other using Micro-Tools™ (Hampton Research). A native crystal from optimized conditions was transferred to a cryoprotectant composed of reservoir solution with 20% (v/v) ethylene glycol prior to data collection. A NaI-derivatized crystal was prepared according to the quick-cryosoaking procedure [23]. A native crystal was soaked for 30 s in a drop containing 0.8 M NaI in the same cryogenic solution used for native crystal data collection. Diffraction data sets from native and NaI-derivatized crystals were collected in-house using a MAR 300 image plate detector and a Rigaku RU200 rotating anode generator. Later on, a 1.15 Å resolution data set was collected at ESRF-Grenoble on beamline ID23. Crystals of form B were picked up directly from the crystallization droplets, mounted in nylon loops and flash-frozen in liquid nitrogen since the 30% MPD in the mother liquor served as a cryoprotectant. The X-ray data collection at 1.45 Å resolution was performed at station ID14, ESRF Grenoble. All the data sets were integrated using *XDS* [24] and scaled using *SCALA* [25] from the *CCP4* package [26]. Data processing statistics for all crystals are shown in Table 1.

**Table 1:** Data collection and refinement statistics.

Data collection statistics	Form A (Native)	Form A (NaI quick soak)	Form A (HR)	Form B
Radiation source	In-house	In-house	ESRF ID23-EH1	ESRF ID14-EH1
Wavelength (Å)	1.5418	1.5418	0.9834	0.9340
Spacegroup	*P*2_1_2_1_2_1_	*P*2_1_2_1_2_1_	*P*2_1_2_1_2_1_	*P*2_1_2_1_2
Cell dimensions *a*, *b*, *c* (Å)	29.83, 44.30, 58.54	29.82, 44.58, 59.03	29.92, 44.58, 59.01	49.43, 78.29, 21.72
Resolution range (Å)	26.58–1.90 (2.00–1.90)	22.85–2.30 (2.42–2.30)	19.00–1.15 (1.21–1.15)	49.43, 78.29, 21.72
Total observations	87402 (12028)	31543 (4535)	92621 (11406)	105291 (12056)
Unique reflections	6485 (907)	3811 (536)	28072 (3995)	15572 (2147)
Completeness (%)	99.5 (98.6)	99.9 (100.0)	98.0 (97.6)	99.5 (97.8)
Redundancy	13.5 (13.3)	7.8 (7.9)	3.3 (2.9)	6.8 (5.6)
*R*_merge_ a	6.7 (20.6)	3.7 (6.3)	8.6 (26.3)	6.8 (24.1)
Average *I*/σ*(I)*	10.2 (3.5)	15.4 (10.4)	9.8 (3.1)	19 (6.4)

Refinement and model statistics	Form A (HR)	Form B
Resolution range (Å)	18.00–1.15	20.00–1.45
Number of reflections used	26611	14757
*R*_work_ (%)^b^ */R*_free_ (%)^c^	12.89 / 15.13	15.97 / 19.57
Average B values		
All atoms (Å^2^)	12.34	14.78
Protein atoms (Å^2^)	10.37	12.48
Thiocyanate atoms (Å^2^)	7.73	-
Ethylene glycol atoms (Å^2^)	12.82	-
MPD atoms (Å^2^)	-	24.76
Ca atoms (Å^2^)	-	15.69
Water atoms (Å^2^)	22.42	23.90
Root mean square deviation from ideality		
Bond lengths (Å)	0.018	0.021
Bond angles (°)	1.595	1.988
Torsion angles (°)	5.869	6.820
Ramachandran analysis		
Favoured regions/ Allowed regions / Outliers (% of residues)	96.7 / 3.3 / 0.0	94.6 / 4.3 / 1.1
No. of atoms		
Protein	731	732
Thiocyanate	3	-
Ethylene glycol	4	-
MPD	-	32
Ca	-	2
Water	144	150

a *R*_merge_ = ∑_h_∑_i_|I_h,i_−<I>_h_|/∑_h_∑_i_ I_h,i_ where <I>_h_ is the mean intensity of the symmetry-equivalent reflections.

b *R*_work_ = ∑_h_∥F_o_|−|F_c_∥/∑_h_|F_o_| where F_o_ and F_c_ are the observed and calculated structure factor amplitudes, respectively, for reflection h.

c *R*_free_ is the *R* value for a subset of 5% of the reflection data, which were not included in the crystallographic refinement.

### Structure determination and refinement

To obtain experimental phases, we first considered using the 11 sulfur atoms present in the native crystals to conduct a sulphur SAD experiment. A highly redundant data set was collected in-house and show good statistics (table 1, Form A native) but was not suitable to give a clear solution to the phase problem. Therefore the quick-cryosoaking method was used. A crystal soaked in a solution containing 0.8 M NaI was collected in-house (table 1, NaI quick soak). The structure of Diedel was solved by SIRAS using SHELXD/E [27] as SAD on the NaI quick soak data set alone also failed to give a clear solution. Initials phases were calculated at 2.3 Å resolution and improved by solvent flattening with DM [26] . Using the program ARP/wARP [28], the majority of the model was correctly automatically built. Incorrectly built remaining residues were manually modeled using Coot [29] and the model was refined in REFMAC [30].

The structure in the crystal form B was solved by molecular replacement with the program AMoRe [31] using the structure solved in crystal form A as starting model. Two strong peaks of electron density were found and were attributed to calcium ions due to the presence of 20 mM CaCl_2_ in the crystallization solution. The first calcium ion is coordinated by Asp 41 and Glu 49 as well as by Glu 25 of a symmetry related molecule. The second calcium ion stands on a crystallographic symmetry axis in the vicinity of Asp 91. None of these residues displays high level of conservation among the Diedel-related molecules.

The structures were analyzed with the program Turbo-Frodo [32]. The statistics on the structure refinement are summarized in Table 1.

### Phylogenetic analyses

Sequences were retrieved from the National Center for Biotechnology Information (NCBI, http://www.ncbi.nlm.nih.gov/) and Flybase (http://flybase.org/blast/) using the sequence retrieval system or/and basic local alignment search tool (BLAST) [33]. Not annotated sequences were found by similarity search and predicted using the gene prediction tools “GENSCAN” (http://genes.mit.edu/GENSCAN.html) and “Eukaryotic GeneMark.hmm” (http://opal.biology.gatech.edu/GeneMark/eukhmm.cgi) and by a manual and careful analysis. Alignments were carried out using clustalW (ref), MUSCLE [34] (www.phylogeny.fr) or COnstraint-Based multiple Alignment Tool (COBALT) [35] (www.ncbi.nlm.nih.gov/tools/cobalt).

Phylogenetic trees were constructed on the basis of amino acid differences using PhyML [34] (www.phylogeny.fr), Fast Minimum Evolution, Neighbor joining and Cobalt Tree [35] (www.ncbi.nlm.nih.gov/blast/treeview). Reliability of the trees was assessed by bootstrapping and comparison between the methods. The number of bootstraps cycles performed for the analysis was 100. The median bootstrap values for the phylogenetic trees were not less than 98%.

### Data deposition

Atomic coordinates and structure factors have been deposited in the RSCB Protein Data Bank under the accession codes 3ZZO (Form A) and 3ZZR (Form B).

## Supporting Information

Text S1 **Protein sequences of analogues of CG11501.**
(DOC)
