# Supplementary material for: Crystal Structure of Diedel, a Marker of the Immune Response of Drosophila melanogaster
Source: PLoS One. 2012 Mar 19;7(3):e33416. doi: 10.1371/journal.pone.0033416 (PMC3307722; doi:10.1371/journal.pone.0033416)
Supplement: Text S1 — Protein sequences of analogues of CG11501. (DOC) [file pone.0033416.s001.doc]

>CG11501_melanogaster

MASPVVSLLLVGICALAFVHVARSECCTSRELVEFKMDRGDCEAVRAIENYPNGCEVTICADGVAQLGAYCGQGSCNIFGCNCDGGCLSGDWSQEFVRRNQQYGIQIIKVTRLPF

>CG43228_melanogaster

MNQLTIAIFWLACINFASSTCCTFAATLHFKIRGGVCGVVGAKSNGTGCKITICPNGEALVGSYCGKAACDVFGCQCIRGCLQGSFAKDFLKKNYIHGIELLGTERTELCFLCQYIPKKIEFEIN

>CG34329_melanogaster

MRAVSVSVLLILVVWVSCFGESGADCCWTKAKLLFTMGTGSCGMVNAKTTKYGCEATVCADGRVLKGTYCGVGSCNIIGCFCRGGCLTGNYGESFVEINNRYQINLISTQMRLANLTDTEA

>GD21438_simulans

MASPVVSSLLLVGIFSLAFVQVARAECCTSRELLEFKMDRGDCAAVRAIENYPHGCEVTICADGVAQLGAYCGKGPCNIFGCNCDGGCLTGDWSQDFVRRNRDYGIQIIKVTRMPL

>GD21443_simulans

MNRLTTAIFWLACINFASSTCCTSAATLHFKIRGGVCGIVGAKSNGTGCKITICPNGEALVGSYCGKAACDVFGCQCIRGCLQGSFAKDFLQKNYIHGIELLGTERTELCFLCQYIPKKIEFEIN

>GD17420_simulans

MRAVSVSVLLILVVWVSCFGESGADCCWTKAKLLFTMGKGSCGMVNAKTTKYGCEATVCADGRVLKGSYCGVGSCNIFGCFCRGGCLAGNYGESFVEINNKYQINLISTQMKLANLTDTEA

>GM12788_sechellia

MAFSVVSSLLLVGICSLACVQVTRAECCTSRELLEFKMDRGDCEAVRAIENYPHGCEVTICADGVAQLGTYCGQGPCNIFGCNCDGGCLTGDWSQDFVRRNRHYGIQIIKVTRMPL

>GM12796_sechellia

MKQLTIAIFWLACINFASSTCCTSAATLHFKIRGGVSGIVGAKSNGTGCKITICPNGEALVGSYCGKAACDVFGCQCIRGCLQGSFAKDFLQKNYIHGIELLGTERTELCFLCQYIPKKIEFEIN

>CG11501c_sechellia

MRAVSVSVLLILVVWVSCFGESGADCCWTKAKLLFTMGKGSCXMVNAKTTKYGCEATVCADGRVLKGSYCGVGSCNIFGCFCRGGCLAGNYGESFVEINNKYQINLISTQMKLANLTDTEA

>GE23853_yakuba

MTSPILTLLLVGICLCFVQVARSECCSSRQEVTYKMDRGDCEDVGGHGDYPLKCEVTICADGVAQVGTYCGQGSCNIFGCNCDGGCLTGEWSDDFVRKNQKYGIEIISVRWIPW

>GE23854_yakuba

MSSPIVTLLLVGICCLSFAQVARSECCTAREVVSYKMDRGDCQDVGGHGDYPLRCEVTICADGVAQVGTFCGQGSCNIFGCHCDGGCLFGEWSEDFARKNQKYGIHIVDVRRIPL

>GE17767_yakuba

MRPVWVPVLLLLVVCVSWFGAADAVCCRTKAKLIFTMGYGSCGMVNAKSTKNGCEVTICPDGRALVGTFCGVGSCNLFGCHCRGGCLSGNFGESFVERNRKYEINLISTQMHLANLTDAVV

>GE23861_yakuba

mkrltiavfwlacinlasttcctsaatlhfkirggvcgvvgaksngtgckiticangealvgsycgraacdvfgcqcirgclqgsfasdflkknyihgiellgtemselcflcqfiprklevain

>GG11665_erecta

MTSPVVTVLLVGIGCLAFVHVARSECCTSREEVKYKMDRGDCEDVGGSGDYPLKCEVTICADGVAQVGTYCGQGSCNIFGCHCDGGCLTGDWSEEFVRKNQAYGIHIVEVRRIPI

>GG19205_erecta

MRAVSASVLLILVVWVSSFGESNADCCWTRAKLIFTMGNGSCGMVNARSTRYGCEITVCADGMVLVGSYCGKGSCNLFGCFCRGGCRRGNFGESFVEINRRYEINLISTQLKMANVTDTEA

>GF15937_ananassae

MRVSVVSALILATCFLAWIDSSEADCCTSMSHLKYTIEGGDCGAVGGSRTSDGCSITICGNGKALVGTFCGRGPCNIFGCACKGGCLSGDFALDFIKNNPGYKIHVTSTVHRS

>GF15938_ananassae

MRLSVVSALILATCFLAWIGSSEAVCCRSKANLKYTIEGGDCGAVGGRRTSDGCTITICGNGRAVVGTWCGRGSCNIFGCACKGGCLSGDFALDFIKNNPGYKIHVTSTVHKP

>GF16013_ananassae

MRLRSVLGVPCAVLVIGVLAQDSAAACCKAQFIRFKTNGFCETVDAIKHEYYAYCETTICADGKRIGKGRYCAQGRCNVFGCNCDGGCRQGDWERSFRNRYPKKQIWFI

>GA11037**_pseudoobscura

MFILPYILVIGLLCLPSKTIAECCRPFPVTFHLAKTNSLLNCEMFGGTQDINYACKSNICGDGMNVWGAWCGQGACNPRGCSCVKGCIEGDPIMNFRLKHGLDNFVYVGPQLENYLYHLTH

>GA26838**_pseudoobscura

MRFLIVLSAMMACTLLLWLPPLAMAVCCRPTRIRFAVMPSIEERNCNTYGGVPDKRQKTCKAAICNNGQPVAGQWCGIGKCNATGCRCKNGCIKPRSDAVRTFLDKNGHSKFVFVRRAPAIVDN

>CG11501c*_pseudoobscura

MGFLAIRALISILAIWLACLVWQASSICCKPTVISFELSVSAEGESCEYFGGTSNEDGSCLASICNDGKAVKNKWCSRGNCDSSGCRCKRGCIRRKFDPVASFNEIHGFLNFLSVSRNVEKPRKFRETEKEHQV

>CG11501d*_pseudoobscura

MRFFIFLPLILAIWLICLSSTTKADCCKPTKIRFELAANNGTHTCETYGGKPGKNYTCKAKICNDGNNVRGTWCGKGRCNPSGCHCKKGCLASGASAIDSFREKHGFFNFIYVGHN

>GL13595**_persimilis

MFILPYILVIGLLCLPSKTIAECCRPFPVTFHLAKTNSLLNCEMFGGTQDINYACKSRICGDGMNVWGAWCGQGACNPRGCSCLKGCIEGDPIMNFRLKHGLDNFVYVGPQLENYL

>GL13594_persimilis

MRFLIVLSAMMACSLLLWLPPLAMAVCCRPTRIRFAVMPSIEERNCNTYGGVPDKRQKTCKAAICNNGQPVAGQWCGIGKCNATGCRCKNGCIKPRSDAVRTFLDKNGHSKFVFVRRAPAIVYN

>CG11501c*_persimilis

MGFLAIRALISILAIWLACLVWQTSSICCKPTVISFELSASAEGESCEYFGGRSNEDGSCLASICNDGKAVKNKWCSRGNCDSSGCRCKRGCIKRQFDPVASFNEIHGFLNFLSVSRNVEKPIKLSLPESQRKERTSGVDMN

>GL13593_persimilis

MRFFIFLPLILAIWLICLSSTTKADCCKPTKIRFELAANNGTHTCETYGGKPGKNYTCKAKICNDGNNVRGTWCGKGRCNPSGCHCKKGCLASGASAIDSFREKHGFFNFIYVGHN

>GK18957_willlistoni

MHFQLLRTVGVLLLILWQSIHVKSICCPRRTLQFKVKSGEDSCFKFSARKISPKVCTMKVCNNGHEPMRRHCGIDSCNFFNCNCMGGCFRGNAAKMFIQSNGGESRFVYAIIK

>GI24242_mojavensis

MKNTSTILLAATLCVLVFVGSARADCCRRTKNVSFTVESGRCKDVGGVNSSISTCTITICADGTRLIGSYCGHYSCNLFGCNCGGGCLKGKWEKNFIERHPKYKIRILSSKWN

>CG11501b*_mojavensis

MKTTTILLALALALAYVGCAQAQCCRRSMVLTYTVGRGRCGDAGGRAGRGGTCSIVICAHGKRKVGTYCGRRSCNIFGCNCGGGCIEGQWQRSFINNNRNQNIRITNTRWIN

>GI22343_mojavensis

MLFLSPLPQWLLLWLGLCSLLLQFRPTKGVCCPSRVIAFQLIDRDDECRLYGAKQSQFGMCTMSICNDGTAVQGTYCGQGPCNIFGCNCENGCRRGNPLQIFQDYYGDYHIRQVHFL

>GI11819_mojavensis

MLQFSCLIAVLGVLWLLVLQAPDASAVCCAEAKVVKYKVLNGQCAAIRGQRSDDGNCVIKICGDGRPLRGTYCGRGSCNLAGCDCDGGCLPGDWEKSLVSDTGIYRVEILEIYWQDLSIFRIPSQSIWDLAASVIGGPKK

>GJ11856_virilis

MAIAMCYFMVLQAPVVQSECCVDAKKLDYTVEDGTCASAGGKASGNGKCVITICGNGEALRGAYCGRGSCNVFGCDCDGGCLGSGWEQTFLQNAAQYKVSLLGWSWTSLSAAERVYEGGLKILDEVKINIG

>GJ14220_virilis

MRFGTSVPSWLLLGLLLQVIVPGAGECCHSKTIAFQLADNEDDCSLYDSKLTKGGVCKMSICDDGSAVEGNFCGQGPCNIFGCNCDGGCRRGDGVQIFRDFYGDYHVRNVHIV

>GJ14221_virilis

MKLALIASLSMALWLLSLSSGATADCCRPSKILYKLANNGKDHNCQTYGGKYHNKETCEKKVCGNGDGIVGTWCGRGKCNPRGCHCRNGCLPGEATASFREKHGYFNFEYVGYA

> XP_001944353.1_Acyrtho

MELYAYQIAIFVVLYLFVSETTSLCCHLPYMQSLPCGIESKSVAEIKLLAPTDERRDVGMPSSVDIITDLASGLCSYSLCAPGYIKKKGVYCATGSCNVFGCNCDGECINENYVDGNLQRLRRESDNINLTYEHLSRASRSTIYLVDKTRKRNTIYTDEKLDSHTPTTNSMTSQVHQ

>CG11501*_Acyrtho

MNSLLCVSVSLLLVLLQSLEINSECCNLQSSVKYTPKGDYTCEEAVPMGQRSLLCLLPRCCVVNHMCHDAKVYPGFYCSSGTCNAVGCNCDGPCIDGDHKTLADHFRLLYPNAEYGYETVSVKNTRAITGFII

> YP_001110948.1_Heliothis_ascovirus

mfrviiffgilvvrqsvsercgyttiafrtkliygvpntpevqrknvgmrcvdfegatynpiqqsygvnsyegickvsvc

gdgkvheghycgigpcnifgsnceggchrgdpvvsfkelyagkvwdvhil

>YP_003422448.1_Pseudaletia_granulovirus

msvvkvivlltvcvlvtseakccrkivvawyasncspysndrsvdnepvlckvkicedgkpnrgyycgkgdcnifgcncd

ggciegygvynfreisgiqrakpiidftdlsiw

> NP_059254.1_Xestia_c_nigum_granulovirus

mlpktghvmvlirlysvrqrqserpilckvkvcndglpnrgfycgkgdcnifgcncdggciqgnaaynfreksglyqaqp

mvdftdlsiw

> YP_762476.1_Spodotera_ascovirus

mnrvgikfvfvvslvvvldifgdrceavcckrtrivwtnpeqgcgkyprefyvnsswwriqrmpdgcvahvcndranrta

cstvgvgkcnifgcncdaravsagdgdalenfkrisgvqnaagplfnykdpttwg

>YP_001649090.1_Helicoverpa_granulovirus

msvvrvivllivsvlvtneakccrklvmswyssdcspyakdqseipilckvkvcndglpnkgffcgkgdcnifgcncdgg

ciegdaaynfreksglyqaqpmvdftdlsiw
